# Prize Essays

**Published:** 1859-01

**Authors:** N. S. Davis

**Affiliations:** Permanent Secretary of the Society


### PRIZE ESSAYS.

At the last annual meeting of the Illinois State Medical Society, two prizes were offered; one of $50, for the best essay, without limiting the choice of subjects; and the other of $20, offered by Dr. J. M. Steele, for the best essay on the Use of Opium in Inflammatory Affections.

Competition for the latter prize is restricted to members of the profession under forty years of age. Both of these offers have been repeated for two or three years, without eliciting any efforts to secure them. We hope it will not be so this year.

Papers designed for either of these prizes should be accompanied by a sealed envelope, containing the name of the writer, and forwarded either to the chairman of the Committee on Prize Essays or to the permanent secretary of the society, on or before the first of May next.

N. S. DAVIS,
*Permanent Secretary of the Society.*

